# Parallel Propagation of *Toxoplasma gondii* In Vivo, In Vitro and in Alternate Model: Towards Less Dependence on the Mice Model

**DOI:** 10.3390/pathogens11091038

**Published:** 2022-09-13

**Authors:** Maria João Gargaté, Anabela Vilares, Idalina Ferreira, Tânia Reis, Susana Martins, Joana Mendonça, Vítor Borges, João Paulo Gomes

**Affiliations:** 1 National Reference Laboratory of Parasitic and Fungal Infections, Department of Infectious Diseases, National Institute of Health Doctor Ricardo Jorge (INSA), Av. Padre Cruz, 1649-016 Lisbon, Portugal; 2 Innovation and Technology Unit, National Institute of Health Doctor Ricardo Jorge (INSA), Av. Padre Cruz, 1649-016 Lisbon, Portugal; 3 Genomics and Bioinformatics Unit, Department of Infectious Diseases, National Institute of Health Doctor Ricardo Jorge (INSA), Av. Padre Cruz, 1649-016 Lisbon, Portugal; 4 Faculty of Veterinary Medicine, Lusófona University, 1749-024 Lisbon, Portugal

**Keywords:** *Toxoplasma gondii*, 3Rs, strain propagation, mice model, in vitro propagation, virulence

## Abstract

*Toxoplasma gondii* is an obligate intracellular protozoan. In pregnant women, it can lead to severe birth defects or intrauterine death of the fetus. Most of what is currently know on cell biology of *T. gondii* comes from studies relying on the RH strain propagated in mice. According to the recommendations concerning the animal welfare, we assayed in vitro/in vivo procedures to replace, or at least reduce, the demanding animal model for strain propagation. We evaluated the genetic and phenotypic stability of the RH strain throughout its parallel continuous propagation in mice, in human foreskin fibroblasts (HFF) and in an alternate fashion of these two procedures. We also assessed the virulence impact on the RH strain after different periods of its long-term propagation strictly in cells. The RH strain completely lost its virulence after long-term passage in HFF. Nevertheless, we obtained a successful outcome with the alternate passaging of the parasite in HFF and in mice as this approach enabled *T. gondii* to maintain the evaluated phenotypic properties, mainly its virulence potential. Also, no genetic changes were observed in genes known to be highly polymorphic or involved in pathoadaptation. In conclusion, the alternate model seems to be a feasible method for *T. gondii* propagation and maintenance, strongly impacting the number of sacrificed mice.

## 1. Introduction

*Toxoplasma gondii* is an obligate intracellular protozoan that infects up to a third of the world’s human population [1]. It is mostly asymptomatic in pregnant women but can lead to severe birth defects or intrauterine death of the fetus. In immunocompromised patients, namely patients undergoing organ transplantation or with AIDS, reactivation of latent infection may lead to life-threatening encephalitis [1]. It is assumed that more than 95% of *T. gondii* strains belong to one of three major clonal lineages, known as types I, II and III, which predominate in North America and Europe [2]. The RH strain and most of other highly virulent strains for mice are classified into lineage I, whereas the non-virulent strains mostly cluster into lineages II or III. Almost everything we currently know about cell biology, immunology and genetics of *T. gondii* has been discovered using the RH strain as a model. This strain was isolated in the USA by Albert Sabin from a 6-year-old boy who died of encephalitis in 1939 [3]. Since then, it has been extensively passaged in mice throughout decades for both research and laboratory purposes. Population genetics and epidemiological studies have indicated that the pathogenesis of *T. gondii* infections depends on the genotypes and clones that circulate in different regions of the world. Also, it has been suggested that the virulence phenotype that different *T. gondii* strains reveal by using the mice model is somehow correlated with disease manifestations in humans [4]. Therefore, it is believed that the determination of *T. gondii* virulence in mice may be invaluable in predicting the potential outcome of human infections.

The legislative regulations introduced by several countries [5,6,7] impacted the development and evolution of the laboratory animal manipulation. In 1959, the book authored by Russell and Burch, entitled *The Principles of Humane Experimental Technique,* has become an essential publication in the science of animal experimentation because it highlights how animal experimentation can be diminished or removed [8]. These authors introduced the *Three Rs concept*, designating the terms Reduction (i.e., decrease of the number of animals used), Refinement (i.e., diminish the incidence or severity of painful or distressing procedures) and Replacement (i.e., substitution of living animals by in vitro techniques, computerized models, etc.), as a main guideline for the responsible use of animals in laboratory experiments. This concept is particularly applicable to the studies involving *T. gondii* given the very high number of mice used in reference laboratories, essentially for strain maintenance and diagnosis purposes. According to the recommendations concerning the animal welfare on behalf of their use in laboratory, we aimed to evaluate the potential of in vitro/in vivo procedures to replace, or at least reduce, the number of sacrificed animals needed for *T. gondii* propagation in reference laboratories. Some studies have already been performed towards the use of different cell lines for *T. gondii* propagation, yielding quite heterogeneous results in terms of the obtained parasite concentration and viability [9,10,11,12,13,14].

In the present study, we focused on a single cell line and opted for performing a long-term 3-way parallel experiment, which contrasts with the approaches of previous studies. In particular, we evaluated the genetic stability and basic phenotypic traits of the virulent *T. gondii* RH strain throughout its parallel propagation in mice, in human foreskin fibroblasts (HFF), and in an alternate fashion of these two procedures, for a period of 10 months.

## 2. Materials and Methods

The RH strain used in the present study has been routinely maintained in mice at the National Reference Laboratory of Parasitic and Fungal infections (NRLPFI) from the Portuguese National Institute of Health (INSA) since 1985. Propagation in mice is routinely performed by syringe intraperitoneal (ip) inoculation of ascitic fluid (AF) at 2-day intervals.

This study was approved by the Ethical Board of INSA, Comissão de Ética para a Saúde (CES-INSA), with the licence number 68.

### 2.1. General Workflow

**Figure 1:**
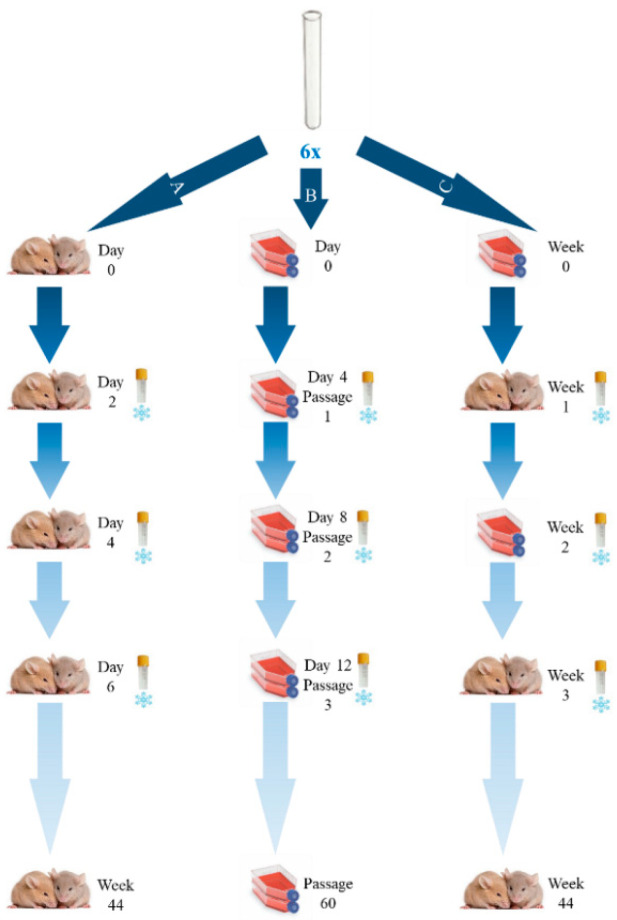
Workflow of RH strain inoculations. Experiment A: continuous mice inoculation, which took place every two days; Experiment B: continuous cell line (Human foreskin fibroblasts) passages, taking place every 72–96 h; Experiment C: intercalate cell line passages and mice inoculation. Before each inoculation in mice, tachyzoites were propagated for two periods of 72–96 h (designated as “week” in the Figure) in the cell line. The study duration was about 44 weeks. Although the experiment B consisted of a continuous passaging of the RH strain exclusively in the cell line, an aliquot taken from the harvesting procedure at week 3, 22 and 44 was used for a mice inoculation in order to evaluate the phenotypic impact after different periods of long-term propagation of *T. gondii* strictly in cells.

We prepared six suspensions of the RH strain obtained from the inoculated mice AF (Figure 1), each containing about 1 × 10^5^ of tachyzoites in 0.2 mL. The concentration of tachyzoites was determined by using a Neubauer chamber under light microscope and its viability was accessed after staining a cover slip with metilen blue at 0.1% (*v/v*). These suspensions were used to simultaneously inoculate two mice for “experiment *A”*, two cell culture flasks for “experiment *B”* and two additional cell culture flasks for “experiment *C”*.

As represented in Figure 1, the experiment A consisted of continuous passages of the RH strain in mice, the experiment B consisted of continuous passages of the RH strain in a cell line, and finally, the experiment C consisted of intercalate passages of the RH strain in the cell line and in mice. This study was developed during a period of approximately 10 months. In order to properly define the passage dynamics involving in vitro culture (Experiments B and C), we set up a preliminary assay to evaluate whether sequential in vitro passaging lead to significant phenotypic alterations in the tachyzoites. We have consistently observed a considerable decrease in the number and motility of the tachyzoites, as well as their shape alteration (through microscopic evaluation) after three passages (72–96 h each). As such, for Experiment B, which is a challenging mice-independent procedure, to avoid the culture loss, whenever a strong decrease in the number of harvested tachyzoites was observed (after approximately 12 days), the aliquot of the immediate previous passage was thawed and was used to proceed the continuous cell line culture. For Experiment C, we opted for a highly conservative approach by designing it with periods of RH passaging in the cell line not exceeding two passages (about one week).

For all experiments, at the harvesting steps (detailed below), a 500 µL aliquot was taken and was frozen at −20 °C. From this large set, 30 samples (10 samples from each experiment A, B, and C), corresponding to one sample per month, were subjected to genetic characterization of the passaged strain by Next Generation Sequencing (NGS) (also detailed below). Additionally, two control mice were injected with DMEM only and two cells culture flasks were used as controls.

### 2.2. Mice Propagation Procedures (Experiments A and C)

We used female CD1 mice with 6–8 weeks old and weight of 16–18 g [Hsd:ICR (CD-1^®^); Harlan Ibérica, Barcelona]. Mice inoculation, maintenance and euthanasia were performed under the standards of INSA, which are in accordance with the Protocol of International Guiding Principles for Biomedical Research Involving Animals, as issued by the Council for the International Organizations of Medical Sciences. Animals were housed in cages and maintained under controlled conditions (21 ± 2 °C, 65–70% humidity) and standard food and water *ad libitum* during the experiments.

A suspension of the RH strain (0.2 mL, corresponding to 1 × 10^5^ tachyzoites) was inoculated in mice by syringe ip inoculation and after 48 h mice were euthanized. Then, tachyzoites were harvested from the peritoneal cavity of infected mice by flushing with 5 mL of sterile phosphate buffer saline (PBS; 0.01 M, pH 7.2), and were centrifuged at 2000 rpm for 10 min at room temperature. The pellets enriched with tachyzoites were recovered with PBS and 0.2 mL of a 1 × 10^5^/mL suspension were ip inoculated in another animal. Following this procedure, brain tissue was harvest from the mouse and observed in the light microscope in order to search for *T. gondii* cysts. Visual inspection was used to identify the phenotypic effect of *T. gondii* RH strain in mice, namely, the observation of the general condition of the animal, the hair condition and the stool consistency. This entire procedure was rigorously followed in experiment A, and, with exception of the timelines, it was also followed in experiment C for the steps enrolling mice inoculation and harvesting.

### 2.3. Cell Culture Propagation Procedures (Experiments B and C)

Human foreskin fibroblasts (HFF-1ATCC—SCRC-1041) were grown in 10 mL of culture medium using 25 cm^2^ flasks (Sarstedt, Nümbrecht, Germany, 831810.002). For maintenance purposes, the HFF was grown in Dulbecco’s Modified Eagle Medium (DMEM) (Gibco, Waltham, MA USA, 42430–25) with 10% of heat inactivated fetal calf serum (FCS) (Gibco, 10270106), penicillin (12 µg/mL), streptomycin (10 µg/mL) (Gibco 15140-122) and (1%) fungizone (Gibco 15290-018) and was incubated in 5% CO_2_ at 37 °C and >80% humidity. Then, when a confluent monolayer was obtained, a DMEM supplemented with FCS 5% was used. Cells were routinely subcultured every 3 days being subjected to trypsination [0.25% trypsin (Gibco 25300-062) with 0.03% EDTA solution] and washing with phosphate-buffered saline (pH = 7.2). 

For the inoculation with the RH strain, after 70% confluence of the cell line, 1 mL of the suspension of tachyzoites (~1 × 10^5^) was added to the cell line followed by an incubation at 37 °C, >80% humidity and 5% CO_2_ for 72–96 h. Two 25 cm^2^ flasks were always simultaneously inoculated. After this period, the medium was aspirated and the cells monolayer was washed with 5 mL of sterile PBS and scraped. The amount and the viability of tachyzoites harvest from the cell culture were determinate with a Neubauer chamber under light microscope (400×) after staining with metilen blue 0.1%. These tachyzoites were then diluted to a final concentration of 1 × 10^5^/mL, constituting the new inoculum either for a new set of two 25 cm^2^ flasks with 70% confluence of HFF cells (Experiment B), or for the inoculation of two mice (Experiment C). From each passage, a 500 µL aliquot was taken and frozen with 1 mL of 50% FBS and 1 mL of 20% DMSO in cryovials with a cooling rate of 1 °C per minute until reaching −80 °C. Cryovials were then placed in a –80 °C freezer or in a liquid nitrogen tank. Whenever the need to use a frozen aliquot arose, the frozen aliquot to be used for inoculation purposes was placed in a water bath at 37 °C with shaking, until completely thawed. In order to identify the phenotypic effect of the *T. gondii* RH strain on the inoculated cells, direct examination with a phase contrast optics microscope (400×) was used. This entire procedure was rigorously followed in experiment B, and, with exception of the timelines, it was also followed in experiment C for the steps enrolling cells inoculation and harvesting.

Although the experiment B consisted of a continuous passaging of the RH strain exclusively in the cell line, an aliquot taken from the harvesting procedure at weeks 3, 22 and 44 was used for an isolated mice inoculation in order to evaluate the phenotypic impact after different periods of long-term propagation of *T. gondii* strictly in cells.

### 2.4. DNA Extraction, PCR and Next Generation Sequencing (NGS) 

RH *T. gondii* DNA was extracted directly from the tachyzoites suspension of AF and from the tachyzoites that were harvest from HFF cells suspension, by using the QIAamp DNA mini kit for tissues (Qiagen, Chatsworth, USA), according to the manufacturer’s protocol.

In order to perform a brief evaluation of the genetic stability of *T. gondii* on the course of the three described experiments, the following loci, which are spread by different *T. gondii* chromosomes (http://toxomap.wustl.edu/verticalmap08_01-2005high.jpg, accessed on 3 January 2021), were enrolled in this study: (i) *Sag*2 (the classical typing gene); (ii) three loci traditionally used for microsatellites-based typing (TgM-A, B18, W35); and, (iii) five polymorphic loci potentially involved in adaptation/virulence (CB21-4, PK1, *Gra*6, *Sag*3, M102). Target regions and PCR primers for all loci are described in Table 1, and are divided in two panels (with and without 5′ adapters for amplicon-based NGS Illumina protocols), according to the amplicon size and the position of the target microsatellites within some amplicons. In order to generate high quality amplicons for subsequent high-throughput amplicon-based NGS, different PCR were conducted. 

Briefly, a multiplex PCR was applied for loci M102, B18 and W35, while *Sag*2, TgM-A, CB21-4, PK1, *Gra*6, *Sag*3 were targeted by independent PCR. All PCR products were visualized in GelRED (Biotarget, Lisbon, Portugal) stained 2% agarose gel electrophoresis. For each sample selected for NGS, PCR products were pooled separately (i.e., with and without Illumina adapters) before being purified and subjected to the Nextera XT DNA Library Preparation protocol (Illumina Inc, San Diego, CA, USA), according to manufacturer’s instructions. Finally, libraries obtained from amplicons with and without Illumina adapters were independently sequenced (2 × 150 bp paired-end reads) using a MiSeq (Illumina) equipment. 

### 2.5. Bioinformatics Analyses

For Single Nucleotide Polymorphisms (SNPs)/indel screening, NGS data was processed using the mapping-based bioinformatics pipeline implemented in INSaFLU (https://insaflu.insa.pt/, accessed on 3 January 2021), which is a web-based platform for amplicon-based NGS data analysis [15]. Briefly, the core bioinformatics steps involved were: (i) raw NGS reads qualityand improvement using FastQC v. 0.11.5; (https://www.bioinformatics.babraham.ac.uk/projects/fastqc, accessed on 3 January 2021) and Trimmomatic v. 0.27 (http://www.usadellab.org/cms/index.php?page=trimmomatic, accessed on 3 January 2021), respectively; and, (ii) reference-based mapping, consensus generation and variant detection using the multisoftware tool Snippy v. 3.2-dev (https://github.com/tseemann/snippy, accessed on 3 January 2021), using a multi-FASTA file with representative sequences of each of the PCR target as reference sequence. Mapping results were inspected and confirmed through visual inspection using the Integrative Genomics Viewer (http://www.broadinstitute.org/igv, accessed on 3 January 2021). 

For in silico microsatellite-size analysis, we took advantage of a previously applied bioinformatics script [16] that allows capturing, directly from NGS reads (after quality improvement with Trimmomatic), the repeats number profile within the microsatellite region. This strategy consists on the extraction and counting microsatellites DNA sequences that are flanked by two conserved small DNA strings. The defined strings for the microsatellite-containing loci are detailed in Table 1.

All raw sequence reads generated in the present study were deposited in the European Nucleotide Archive (ENA) (BioProject PRJEB34235). Detailed ENA accession numbers are described in Supplementary Table S2 (DOI: 10.6084/m9.figshare.20765722).

## 3. Results

**Figure 2:**
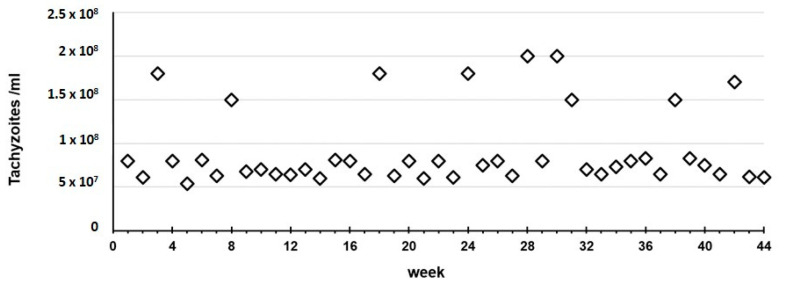
Experiment A: Continuous propagation of the RH strain in mice. The squares represent the tachyzoites concentration harvested during the 10-month study period.

Throughout the entire 10-month study, in experiment A we observed that the RH strain always killed the mice in two days and presented tachyzoites-rich ascites, with a median of 7.50 × 10^7^ tachyzoites/mL (mean of 9.15 × 10^7^/mL, SD 4.30 × 10^7^/mL) recovered in the harvest procedure (Figure 2). 

**Figure 3:**
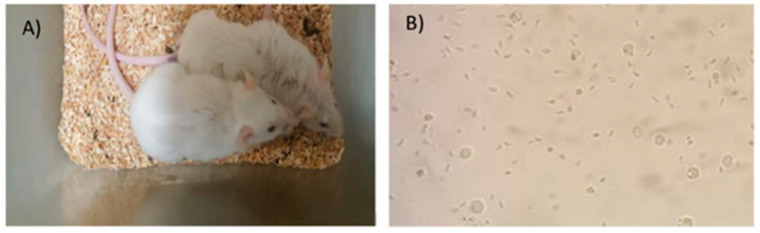
Phenotypic evaluation on the course of experiment A. Panel (**A**) shows the phenotypic effect of RH strain propagation in mice, where mice can be observed totally prostrate in a corner of the cage and with bristly hair; Panel (**B**) shows tachyzoites with a thin shape and motile rich ascites (40× magnification). Image source: NRLPFI.

During these two days, the animals showed great prostration, completely bristly hair and soft stools (Figure 3). In the necropsy of the more than 200 mice enrolled in experiment A, no *T. gondii* cysts were observed in mice brain tissues.

**Figure 4:**
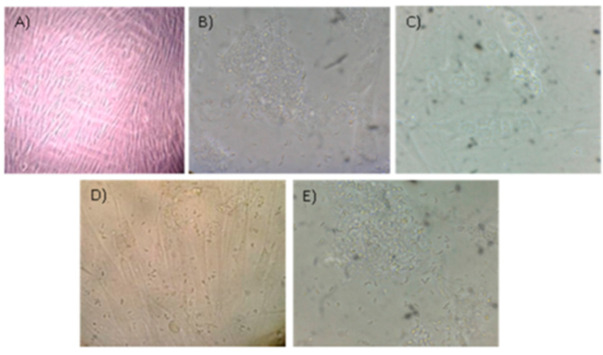
Phenotypic evaluation on the course of experiment B. Phenotypic effect of RH strain inoculation in HFF (40× magnification). Panel (**A**)—HFF monolayer; Panel (**B**)—Tachyzoites replicated in the cells within 6 to 9 h; Panel (**C**)—Tachyzoites formed rosettes with the apical ends directed towards parasitophorous vacuole membrane within 24 h; Panel (**D**)—Most of HFF are infected and some of them detached from the flask within 72 h; Panel (**E**)—Monolayer is destroyed and a large number of tachyzoites are in the supernatant within 96 h. Image source: NRLPFI.

Regarding experiment B, we observed an increase in the number of RH strain tachyzoites inside the cells within 6 to 9 h, and after approximately 24 h parasites formed rosettes with the apical ends directed towards the parasitophorous vacuole membrane and some of them were dispersed in the cytoplasm of the host cell. After 72 h, most of the cells were infected. After 96 h, the monolayer was destroyed and a large number of tachyzoites could be observed in the supernatant (Figure 4).

**Figure 5:**
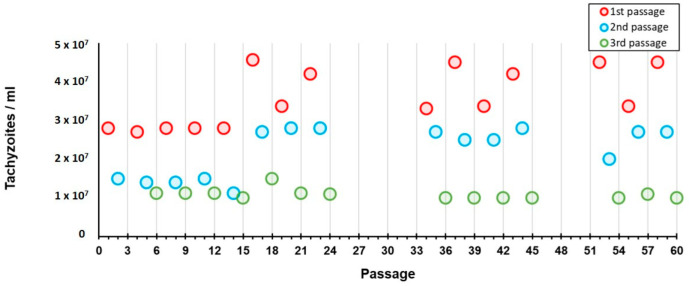
Experiment B: Continuous propagation of the RH strain in a cell line (HFF). The circles represent the tachyzoites concentrations that were harvested during the study period. The different colors represent the three passages within each cycle of ~12 days.

We also observed that, in each cycle of three passages (±12 days), the amount of tachyzoites that were harvested in each passage progressively decreased, from a median of 3.36 × 10^7^/mL (3.58 × 10^7^/mL, SD 7.16 × 10^6^/mL) in the first passage of each cycle, to a median of 1.05 × 10^7^/mL (mean of 1.08 × 10^7^/mL, SD 1.28 × 10^6^/mL) in the last passage (Figure 5). Therefore, in order to avoid losing the strain, the aliquot of the immediate previous passage (i.e., second passage) was thawed and used for the subsequent inoculation. This yielded a highly homogeneous fluctuation dynamic of the tachyzoites recovery during the entire experiment B period (Figure 5). Also, throughout each of these three-passages cycles, the size of the tachyzoites diminished and their shape became rounded, although these phenotypes were hardly seen after the first passage.

We observed that the virulence of the RH strain after long periods of passages in the cell line strongly decreased. In fact, after different periods of long passaging in HFF, the two mice that were inoculated with the harvested tachyzoites did not die in short periods of time. One of them was euthanized for observation after seven days and the other lived for about 11 months. The ones that were euthanized showed no tachyzoites in the AF. 

**Figure 6:**
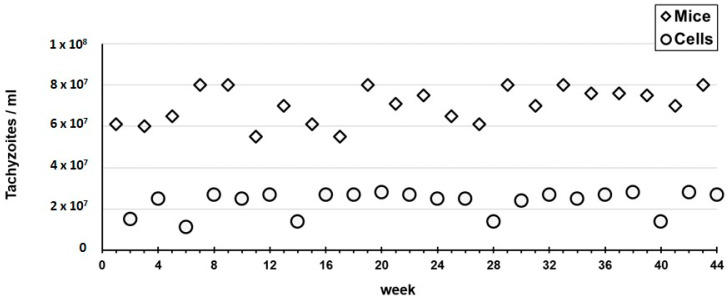
Experiment C: intercalate passages of the RH strain in the cell line and in mice. The circles and squares represent the tachyzoites concentration that were harvested from the cell line (circles) and mice (squares) throughout the 44 weeks.

Finally, regarding experiment C, during the entire 10-months period we observed a phenotypic profile of tachyzoites that was similar to the one described above for the first passage within each cycle of three passages of experiment B (i.e., timelines, rosettes formation and tachyzoites shape). Only sporadic and modest alterations were seen when compared with the ones observed in the tachyzoites from the mice ascites. We observed quite homogeneous values of the tachyzoites concentration that were harvested from the mice (median 7.05 × 10^7^, mean of 7.03 × 10^7^/mL, SD 8.43 × 10^6^) (Figure 6), which were in the same range as the ones observed in experiment A. For the harvested tachyzoites from the cell line, the values obtained (median 2.60 × 10^7^/mL, mean of 2.35 × 10^7^/mL, SD 5.50 × 10^6^/mL) (Figure 6) fitted the interval observed for the three passages within each 12-day cycle of experiment B. 

**Figure 7:**
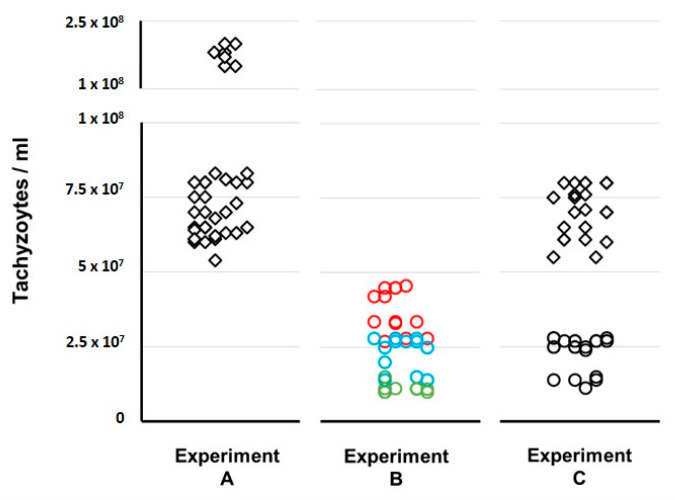
Comparison of tachyzoites yield between the three propagation experiments. For experiments A and C, each value reflects the tachyzoites concentration that was harvested each week, while, for experiment B, each value reflects the tachyzoites concentration obtained after the first (red circles), second (blue circles), and third passage (green circles) within each cycle of three passages (~12 days). Circles and squares represent concentration values obtained from harvested cells and mice, respectively.

For better visualization purposes, Figure 7 shows the comparison of tachyzoites yield between the three propagation experiments. For Experiment C, we observed that, after each period of 72–96 h in the HFF cell line, the tachyzoites showed a mild virulence decreased phenotype in mice as they caused mice death only after about seven days. Curiously, we observed no obvious decrease in the number of tachyzoites (Figure 7) that were recovered in the ascites, when compared with experiment A.

Finally, regarding the evaluation of potential genomic changes arising throughout propagation, which enrolled ten samples (about one per month) from each “experiment” and targeted nine genome loci, we observed no SNP/indels both chronologically (for a given experiment) and between experiments. Also, for microsatellite-harboring loci (B18, W35, TgM A and CB21-4), we observed the same consensus microsatellite length (and profile) for all samples. Of note, the consensus lengths retrieved by our NGS/Bioinformatics-based approach for the RH strain matched the ones previously reported [17] by traditional microsatellites-based analyses relying on electrophoresis-based DNA sizing.

## 4. Discussion

The maintenance of reference strains, namely the RH, in a National Reference Laboratory (NRL) is imperative considering that the Sabin-Feldedman dye test, which is the Gold Standard among serological tests, requires fresh viable tachyzoites. While parasite propagation is mandatory for antigen production, it is also useful for tachyzoites enrichment for typing purposes, and can additionally provide vital information in studies focusing *T. gondii* lifecycle (and its particular stages), host invasion, host–parasite interactions, and host behaviour [18,19,20,21]. Although some of these goals (e.g., antigen production) may be achieved by using cell-lines for tachyzoites’ propagation [9,10,11,13], the animal model remains crucial for studying the natural infection dynamics and parasite dissemination, and is required to confirm findings after initial in vitro investigations [22]. Also, the results of the studies using different cell lines were quite heterogeneous as different approaches were used and, in several cases, a single cell line was evaluated, which hampers the identification of the best cell line for each purpose. Furthermore, the in vivo model has a vital role in a NRL because it is the gold standard method for the isolation of *T. gondii* strains in biological samples or body fluids [23] constituting, for instance, the reference methodology in pre- and post-natal diagnosis of toxoplasmosis. However, this in vivo procedure frequently involves the need to sacrifice hundreds of mice per year, and is also extremely laborious, requiring complex facilities and human skills either for the isolation of strains or for their maintenance process. The legislative regulations and the ethical measures that have been arising regarding the use of animals in the laboratory [5,6,7] push the researchers towards the use of alternative approaches to diminish the animal manipulation. As such, on behalf of our role as the reference laboratory for toxoplasmosis at the Portuguese NIH, we aimed to investigate if we can replace, or at least reduce, the demanding animal model for strain propagation by using “feasible” methodologies. Therefore, we enrolled a 3-way parallel approach consisting of continuous passages of the RH strain in mice (Experiment A), in a cell line-HFF (Experiment B), and intercalate passages of the RH strain in the cell line and in mice (Experiment C).

In order to compare the outcomes of each propagation experiment, we started to evaluate the tachyzoites capacity to maintain an active multiplication in the three experiments. As expected, throughout continuous propagation in mice (Experiment A) we always obtained tachyzoites-rich ascites with values for tachyzoites concentration in the harvesting processes (median of 7.50 × 10^7^/mL) that were up to 7.5-fold higher than the ones obtained for the experiment enrolling the exclusive propagation in the cell line (Experiment B). Curiously, in Experiment C, which enrolled 46 passages in the cell line intercalated with 14 passages in mice, we faced a very encouraging scenario. In fact, we obtained: (i) tachyzoite-rich ascites with harvesting values (median of 7.05 × 10^7^/mL) similar to the ones obtained in Experiment A; and (ii) tachyzoites yields in the multiples harvesting processes of the cell line (median of 2.60 × 10^7^/mL) that were about 2.5-fold higher than the ones obtained at the end of each cycle in Experiment B (Figure 6 and Figure 7). On the one hand, this suggests that the tachyzoites capacity to maintain an active multiplication in a cell line throughout continuous passaging decreases over time (as we observed a ~3-fold decrease from the first to the third passage within each cycle of ~12 days in Experiment B (Figure 5). Nevertheless, our data show that this capacity seems to be renewed when, after short periods in a cell line (e.g., one week, Experiment C), the tachyzoites are transferred to mice.

A second evaluation consisted of assessing potential phenotypic alterations, such as tachyzoites shape and motility, upon propagation of the parasite in a cell line. Contrasting to Experiment A, for which we observed tachyzoite-rich ascites with tapered shape and with motility, the continuous propagation of tachyzoites in the cell line lead to a decrease in their size and to an alteration of its shape, which was notably seen at the end of each 12-days cycle. This was consistently observed for all 12-days cycles of Experiment B. Noteworthy, for Experiment C, minor phenotypic changes were observed in the microscopic observation of tachyzoites harvested from the cell line, and a typical phenotype could be seen in the mice ascites.

We also evaluated the capacity to maintain a virulence phenotype in mice. Regarding Experiment A, we observed that the RH strain always killed the mice in two days, as is routinely observed in our laboratory when dealing with this strain. This is concordant with what is expected for *T*. *gondii* genotype I strains, which are usually responsible for lethal infections in mice, while types II and III are significantly less virulent [18]. Several studies showed [24,25] that a prolonged passage of the type I RH strain in mice leads to an attenuation of tissue cysts / bradyzoites formation. Although this constitutes a non-consensual topic, we observed that, in agreement with several studies [26], our RH strain lost its ability of cystogenesis possible due to the prolonged and uninterrupted mice passages in our lab since 1985. For Experiment B, we also evaluated the impact of the tachyzoites long-term passaging in the HFF cell line at three time points (at week 3, 22 and 44) after the first inoculation, and observed that the RH strain completely lost its virulence. In fact, when we inoculated the harvested parasite into two mice and euthanized one of them (the other died of old age) for observation, there were neither tachyzoites in its ascite nor cysts in the brain. In Experiment C, we observed an intermediate scenario, as some virulence decreased phenotype in mice was observed after ~1 week in cell culture (throughout the entire study period) because RH strain consistently killed the mice after about seven days and not in two days as in Experiment A. This strongly suggests that, although a continuous passaging in cell lines may be useful for some specific proposes (e.g., antigen production), this approach cannot be successfully used for maintaining *T. gondii* strains for other research purposes as they unequivocally loose the virulence phenotype. Nevertheless, hybrid approaches such as the one here represented in Experiment C seem to have the potential to overcome the virulence issue, which is crucial in most *T. gondii* reference laboratories.

Finally in order to assess if the propagated RH strain underwent genomic changes throughout propagation (regardless the passaging experiment), we conducted a humble genomic evaluation focused on both traditional typing loci and selected polymorphic loci that are genome-dispersed and are believed to play a role in adaptation. Whereas the hypothetical detection of genetic alterations throughout the study could suggest the putative inadequacy of using the selected approaches for strain maintenance, we observed no genetic changes throughout each experiment and between experiments. Still, no definitive conclusions about genetic stability can be taken, given the limited extent of the *T. gondii* genomic regions that were surveyed. Nevertheless, broader evaluations at the genomic level are very challenging, considering both the large genome size (~65 Mb) and the high complexity of the 14 chromosomes of *T. gondii*. Genome-wide surveys would make this evaluation impractical and cost prohibitive. Of note, in this study, we unprecedentedly applied a robust NGS/bioinformatics approach for in silico extraction of the microsatellite size for three loci (TgM-A, B18, W35) traditionally used in microsatellites-based *T. gondii* genotyping and that were among the several loci analyzed in this study. This technological innovation constitutes a proof-of-concept that the traditional microsatellites analyses (based on laborious and error-prone electrophoresis-based DNA sizing) might be straightforwardly transfer to more robust NGS/bioinformatics-based methodologies, while keeping backwards compatibility with “historical” typing data.

Besides the above cited need for a challenging broader evaluation at genomic level, some other limitations of this study may be referred, namely: (i) no other cell lines were evaluated in these 3-way parallel propagation approach; (ii) the conclusions obtained with the largely used RH strain shall not be blindly extended to other *T. gondii* strains since it is known that different genotypes display dissimilar phenotypes; and (iii) the present study did not evaluate the potential impact of the tested long-term propagation approaches on other phenotypes such as the host behaviour, which has been the focus of recent studies [20,21].

## 5. Conclusions

As concluding remarks, the results of the present study demonstrate the feasibility of using tachyzoites propagation approaches based on alternate passages on mice and cell lines, strongly impacting the number of sacrificed mice. This would enable laboratories to maintain and propagate *T. gondii* strains and use them for virtually many purposes, as not only the replication capacity, shape and motility of tachyzoites seems to be maintained, but also the virulence appears not to be obliterated. Finally, although a humble genomic survey was performed, no apparent genetic alterations were observed as a consequence of in vitro propagation.

## Figures and Tables

**Table 1:** Loci, amplicon length and primers used for PCR.

**Locus**	**Chr**	**Primer Foward (5′-3′)**	**Primer Reverse (5′-3′)**	**Box 1 (5′-3′) ^c^**	**Box 2 ^c^ (5′-3′)**	**Amplicon Length (bp)**
**Pk 1**	VI	**FI** TCA TCG CTG AAT CTC ATT GC	**RI** CGC AAA GGG AGA CAA TCA GT	NA	NA	873
	
**GRA 6**	X	**FI** TTT CCG AGC AGG TGA CCT	**RE** TCG CCG AAG AGT TGA CAT AG	NA	NA	314
	
**Sag2 5′**	VIII	**F4E** GCT ACC TCG AAC AGG AAC AC	**R4E** GCA TCA ACA GTC TTC GTT GC	NA	NA	305
	
**Sag2 3′**	VIII	**F3E** TCT GTT CTC CGA AGT GAC TCC	**R3E** TCA AAG CGT GCA TTA TCG C	NA	NA	297
	
**SAG 3**	XII	**FI** TCT TGT CGG GTG TTC ACT CA	**RI** CAC AAG GAG ACC GAG AAG GA	NA	NA	211
	
**M 102**	VIIa	**F** GAG CGA CGC CCG TAT GAT AAG G	**R** CGC GCT GAG AAG CTG ACA TAC AG	NA	NA	427
	
**CB21-4**	III	**F** CCA GGT GTT TCG ATA TTG AT	**R** GCC TGT GTG GTG TTC GAA TC	TACGCATACA	GTACATTCTT	469
	
**TgM-A**	X	**F**^a^ GGC GTC GAC ATG AGT TT CTC	**R**^b^ TGG GCA TGT AAA TGT AGA GAT G	CGTGTTTCCA	TTTGTAAGTC	207
**B18**	VIIa	**F**^a^ TGG TCT TCA CCC TTT CAT CC	**R**^b^ AGG GAT AAG TTT CTT CAC AAC GA	TGCCTGTAGC	GGATTCCGCA	160
**W35**	II	**F**^a^ GGT TCA CTG GAT CTT CTC CAA	**R**^b^ AAT GAA CGT CGC TTG TTT CC	TCTTGGCTTT	GTGTCGCTGT	248

^a^ Primers with adapters—TCGTCGGCAGCGTCAGATGTGTATAAGAGACAG; ^b^ Primers with adapters—GTCTCGTGGGCTCGGAGATGTGTATAAGAGACAG; ^c^ Sequences contiguously flanking each side of the tandem repeat region that are used for in silico extraction of the microsatellite size; NA—not applicable. ToxoDB-based ID of the loci analyzed in the present study is presented in Supplementary Table S1.

## Data Availability

The sequence data presented in this study are openly available at BioProject PRJEB34235. European Nucleotide Archive (ENA).

## Supplementary Materials

The following supporting information can be downloaded at https://www.mdpi.com/article/10.3390/pathogens11091038/s1, Table S1: ToxoDB-based ID of the loci analysed in the present study, based on the annotations of frequently used *T. gondii* reference strains. Table S2: BioProject PRJEB34235. European Nucleotide Archive (ENA) accession numbers.
