# Supplementary material for: Parallel Propagation of Toxoplasma gondii In Vivo, In Vitro and in Alternate Model: Towards Less Dependence on the Mice Model
Source: Pathogens. 2022 Sep 13;11(9):1038. doi: 10.3390/pathogens11091038 (PMC9502748; doi:10.3390/pathogens11091038)
Supplement: Supplementary file 1 [file pathogens-11-01038-s001.zip › pathogens-1897549-supplementary.pdf]

Table S1. ToxoDB-based ID of the loci analysed in the present study, based on the annotations of frequently used T. gondii reference strains.

LOCI / STRAIN *	Sag2	CB21-4	PK1	L363	SAG1	GRA6	SAG3	M102
GT1	TGGT1_chrVIII	TGGT1_chrIII	TGGT1_chrVI	TGGT1_chrVIIb	TGGT1_chrVIII	TGGT1_chrX	TGGT1_chrXII	TGGT1_chrVIIa
ME49	TGME49_chrVIII	TGME49_chrIII	TGME49_chrVI	TGME49_chrVIIb	TGME49_chrVIII	TGME49_chrX	TGME49_chrXII	TGME49_chrVIIa
VEG	TGVEG_chrVIII	TGVEG_chrIII	TGVEG_chrVI	TGVEG_chrVIIb	TGVEG_chrVIII	TGVEG_chrX	TGVEG_chrXII	TGVEG_chrVIIa

* The ID information for the T. gondii RH strain is lacking for several genes.

Table S2. BioProject PRJEB34235 European Nucleotide Archive (ENA) accession numbers.

SAMPLE ID ENA	BioProject	READS ENA ACCESSION
Tg_RH_1_A	PRJEB34235	ERR3505173
Tg_RH_2_A	PRJEB34235	ERR3505211
Tg_RH_3_A	PRJEB34235	ERR3505219
Tg_RH_4_A	PRJEB34235	ERR3505218
Tg_RH_5_A	PRJEB34235	ERR3505214
Tg_RH_6_A	PRJEB34235	ERR3505175
Tg_RH_7_A	PRJEB34235	ERR3505196
Tg_RH_8_A	PRJEB34235	ERR3505188
Tg_RH_9_A	PRJEB34235	ERR3505191
Tg_RH_10_A	PRJEB34235	ERR3505210
Tg_RH_11_A	PRJEB34235	ERR3505195
Tg_RH_12_A	PRJEB34235	ERR3505202
Tg_RH_13_A	PRJEB34235	ERR3505183
Tg_RH_14_A	PRJEB34235	ERR3505208
Tg_RH_15_A	PRJEB34235	ERR3505201
Tg_RH_16_A	PRJEB34235	ERR3505162
Tg_RH_17_A	PRJEB34235	ERR3505161
Tg_RH_18_A	PRJEB34235	ERR3505169

Tg_RH_19_A	PRJEB34235	ERR3505213
Tg_RH_20_A	PRJEB34235	ERR3505174
Tg_RH_21_A	PRJEB34235	ERR3505198
Tg_RH_22_A	PRJEB34235	ERR3505160
Tg_RH_23_A	PRJEB34235	ERR3505178
Tg_RH_24_A	PRJEB34235	ERR3505205
Tg_RH_25_A	PRJEB34235	ERR3505204
Tg_RH_27_A	PRJEB34235	ERR3505171
Tg_RH_28_A	PRJEB34235	ERR3505212
Tg_RH_30_A	PRJEB34235	ERR3505203
Tg_RH_31_A	PRJEB34235	ERR3505159
Tg_RH_32_A	PRJEB34235	ERR3505166
Tg_RH_1_NA	PRJEB34235	ERR3505163
Tg_RH_2_NA	PRJEB34235	ERR3505189
Tg_RH_3_NA	PRJEB34235	ERR3505179
Tg_RH_4_NA	PRJEB34235	ERR3505206
Tg_RH_5_NA	PRJEB34235	ERR3505168
Tg_RH_6_NA	PRJEB34235	ERR3505190
Tg_RH_7_NA	PRJEB34235	ERR3505164
Tg_RH_8_NA	PRJEB34235	ERR3505172
Tg_RH_9_NA	PRJEB34235	ERR3505167
Tg_RH_10_NA	PRJEB34235	ERR3505184
Tg_RH_11_NA	PRJEB34235	ERR3505182
Tg_RH_12_NA	PRJEB34235	ERR3505197
Tg_RH_13_NA	PRJEB34235	ERR3505170
Tg_RH_14_NA	PRJEB34235	ERR3505165
Tg_RH_15_NA	PRJEB34235	ERR3505199
Tg_RH_16_NA	PRJEB34235	ERR3505216
Tg_RH_17_NA	PRJEB34235	ERR3505215
Tg_RH_18_NA	PRJEB34235	ERR3505193
Tg_RH_19_NA	PRJEB34235	ERR3505186
Tg_RH_20_NA	PRJEB34235	ERR3505177
Tg_RH_21_NA	PRJEB34235	ERR3505209
Tg_RH_22_NA	PRJEB34235	ERR3505185

Tg_RH_23_NA	PRJEB34235	ERR3505194
Tg_RH_24_NA	PRJEB34235	ERR3505192
Tg_RH_25_NA	PRJEB34235	ERR3505180
Tg_RH_26_NA	PRJEB34235	ERR3505176
Tg_RH_27_NA	PRJEB34235	ERR3505181
Tg_RH_28_NA	PRJEB34235	ERR3505187
Tg_RH_30_NA	PRJEB34235	ERR3505217
Tg_RH_31_NA *	PRJEB34235	ERR3505200
Tg_RH_32_NA *	PRJEB34235	ERR3505207

* RH positive controls
